# Outcomes after Surgical Treatment of Oesophagogastric Cancer with Synchronous Liver Metastases: A Multicentre Retrospective Cohort Study

**DOI:** 10.3390/cancers16040797

**Published:** 2024-02-16

**Authors:** Sander J. M. van Hootegem, Carlo A. de Pasqual, Simone Giacopuzzi, Elke Van Daele, Hanne Vanommeslaeghe, Johnny Moons, Philippe Nafteux, Pieter C. van der Sluis, Sjoerd M. Lagarde, Bas P. L. Wijnhoven

**Affiliations:** 1 Department of Surgery, Erasmus University Medical Center, 3000 CA Rotterdam, The Netherlands; s.vanhootegem@erasmusmc.nl (S.J.M.v.H.);; 2 General and Upper GI Surgery Division, University Hospital of Verona, 37134 Verona, Italy; 3 Department of Gastrointestinal Surgery, Ghent University Hospital, B-9000 Ghent, Belgium; 4 Department of Thoracic Surgery, University Hospitals Leuven, 3000 Leuven, Belgium

**Keywords:** oesophageal cancer, gastric cancer, liver metastases, oligometastasis, surgery, ablation

## Abstract

**Simple Summary:**

Around 10–12% of patients present with oligometastatic disease (OMD) from oesophageal or gastric cancer (OGC). Potential curative treatment is debated in these patients, especially when located in the liver. The aim of this study was to describe the outcomes of patients who underwent surgical treatment of the primary tumour together with local treatment of synchronous liver metastases. We report a 5-year survival of 30%, but disease recurred in 80% of patients. Patients with a solitary liver metastasis may have the best prognosis, but more data are needed to optimise patient selection for curative treatment.

**Abstract:**

Approximately 10–12% of patients with oesophageal or gastric cancer (OGC) present with oligometastatic disease at diagnosis. It remains unclear if there is a role for radical surgery in these patients. We aimed to assess the outcomes of OGC patients who underwent simultaneous treatment for the primary tumour and synchronous liver metastases. Patients with OGC who underwent surgical treatment between 2008 and 2020 for the primary tumour and up to five synchronous liver metastases aiming for complete tumour removal or ablation (i.e., no residual tumour) were identified from four institutional databases. The primary outcome was overall survival (OS), calculated with the Kaplan–Meier method. Secondary outcomes were disease-free survival and postoperative outcomes. Thirty-one patients were included, with complete follow-up data for 30 patients. Twenty-six patients (84%) received neoadjuvant therapy followed by response evaluation. Median OS was 21 months [IQR 9–36] with 2- and 5-year survival rates of 43% and 30%, respectively. While disease recurred in 80% of patients (20 of 25 patients) after radical resection, patients with a solitary liver metastasis had a median OS of 34 months. The number of liver metastases was a prognostic factor for OS (solitary metastasis aHR 0.330; *p*-value = 0.025). Thirty-day mortality was zero and complications occurred in 55% of patients. Long-term survival can be achieved in well-selected patients who undergo surgical resection of the primary tumour and local treatment of synchronous liver metastases. In particular, patients with a solitary liver metastasis seem to have a favourable prognosis.

## 1. Introduction

In Western countries, 40 to 50% of patients with oesophageal or gastric cancer (OGC) have synchronous distant metastases [1,2]. Metastatic oesophagogastric cancer has a poor prognosis of 4 to 6 months [3,4]. Palliative chemotherapy improves the quality of life and may prolong median survival up to 12 months [3,5]. In approximately 10 to 12% of patients, oligometastatic disease (OMD) is diagnosed upon presentation [6]. The recent OMEC project established organ-specific definitions of OMD, but until then, OMD had been defined as up to five metastatic lesions limited to one site, which was also used in the AIO-FLOT3 trial [7,8]. The role of surgery in this subgroup has been a topic of debate, especially for patients with liver metastases.

The REGATTA trial [9], the first randomised controlled trial (RCT) testing whether gastrectomy after adjuvant chemotherapy improves survival in gastric cancer patients with a single non-curable site, failed to show improvement of OS. However, patients with limited-metastatic gastric cancer who responded to induction chemotherapy had a median survival of 22.9 months following resection of the primary tumour and metastatic site in the German AIO-FLOT3 trial [10]. Although promising, only 11 patients (18.3%) in the limited-metastatic group had liver metastases.

For gastric cancer, while no high-level evidence supports surgical treatment, several retrospective studies reported long-term survival in selected patients with liver metastases. A recent meta-analysis comprising 55 studies reported significantly improved OS after surgical resection of the liver metastases, particularly in patients with a solitary lesion [11]. However, the studies included were small series from East Asia and only 13 studies included western patients. The majority of these studies were subject to substantial heterogeneity, including both metachronous and synchronous metastatic disease, and evaluated a limited number of patients treated over a long time period, often without the addition of systemic therapy. In the last decade, many improvements in staging, patient selection, and surgical techniques have taken place, while the implementation of chemo(radio)therapy and targeted therapy has improved oncological outcomes.

In contrast to gastric cancer, data on surgical treatment of liver metastases are scarce for oesophageal cancer. Regardless of the liver being one of the most common sites of dissemination, evidence is limited to a few case series [12,13,14,15]. The role of surgery in patients with OMD of the liver from OGC remains controversial, particularly in the context of multimodality treatment. Given the limited number of patients treated in a single institution, a multicentre study is needed. We aimed to describe the survival and postoperative outcomes of patients with synchronous liver metastases from OGC who underwent surgery aimed at complete tumour removal and/or ablation.

## 2. Materials and Methods

This was a multicentre retrospective cohort study. Before initiation, approval was obtained at the Erasmus MC (MEC-2020-0466) and in each participating centre according to local regulations. Informed consent was not required for this study under Dutch legislation.

### 2.1. Patients

Institutional databases were screened for all patients surgically treated for OGC with synchronous liver metastases (cT1-4N0-3M1HEP) between the period of January 2008 and December 2020. Patients were included if they underwent resection of the primary tumour with simultaneous treatment of liver metastases aimed at complete tumour removal/ablation (i.e., no residual tumour) and met the following criteria: (1) ≤ 5 liver lesions; (2) no evidence of extrahepatic metastatic sites; (3) metastasectomy and/or ablation (microwave or radiofrequency) of the liver metastases was performed; and (4) no second active malignancy. For inclusion, both preoperative diagnosis of liver metastases and detection at the time of planned surgery were permitted.

### 2.2. Staging, Treatment and Follow-Up

Clinical staging was performed in accordance with national guidelines and AJCC staging guidelines. For all centres, this included endoscopy with biopsies and computed tomography (CT) of the chest and abdomen. Endoscopic ultrasonography was used when indicated for the assessment of nodal metastases or extent of the primary tumour (junctional cancers to assess infiltration of the oesophagus and distal gastric cancers to assess infiltration of the duodenum). Tumours located at the oesophagus and junction (Siewert II and III) were routinely staged with ^18^FDG-PET since 2013 and, in the case of extensive lymph node involvement, from 2008 onwards. Patients with a tumour located in the stomach underwent a staging laparoscopy and, from 2016 onwards, an ^18^FGD-PET scan in the case of advanced disease (>cT3 or cN+). TNM categories were reported in accordance with the 8th edition of the AJCC manual of cancer staging [16].

Before proceeding to surgery, patients underwent response evaluation after neoadjuvant therapy and were discussed within the local multidisciplinary team. The surgical techniques used have been described in previous studies [17,18]. In all patients treated with ablation, complete necrosis of the liver metastases was confirmed by either an intraoperative ultrasound, a postoperative CT scan, or both.

After surgical resection, patients were seen every 3–6 months in the first 2 years and at 6-month intervals up until 5 years. Follow-up visits included patient history, physical examination, and routine scanning, either FDG-PET or CT, depending on the tumour location. Further investigations were performed on an individual basis.

### 2.3. Outcomes

The primary outcome was overall survival (OS), defined as the time between the date of surgery and death or last follow-up. Secondary outcomes were disease-free survival (DFS) and postoperative outcomes including complications, 30-day mortality, rate of readmission, and length of hospital stay. DFS was defined as the time between the date of surgery and recurrence, death, or last follow-up.

### 2.4. Statistical Analysis

Categorical variables were reported as count and percentage. Distribution of continuous variables was reported as median with interquartile range (IQR) when non-parametric and mean with standard deviation (SD) when parametric. Median follow-up time was calculated with the reverse Kaplan–Meier method. Survival curves were obtained using the Kaplan–Meier method and differences were tested with the log-rank test. Cox regression analysis was performed to assess which factors were associated with OS. Variables with a *p*-value < 0.2 in univariable analysis were included in the multivariable analysis. The threshold for significance was set at *p* < 0.05 (two-sided). Analyses were performed using SPSS version 28.0 (IBM Corp, Armonk, NY, USA) and the survival and survminer packages in R version 4.3.2 (R Core Team, R Foundation for Statistical Computing, Vienna, Austria).

## 3. Results

### 3.1. Patients

Thirty-eight patients were identified from four institutional databases. After excluding seven patients due to benign liver disease on histopathology (*n* = 1), metachronous disease (*n* = 3), or no surgical therapy (*n* = 3), 31 patients were included for analysis. The median age was 62 years [IQR 53–68] and 81% were male. In six patients, liver metastases were diagnosed during surgery, of which four patients received no neoadjuvant therapy. Twenty patients (65%) had a solitary liver lesion and, in 27 patients (90%), metastatic disease was confined to one liver lobe. All patient and tumour-related characteristics are shown in Table 1.

Twenty-six patients (84%) received neoadjuvant therapy, with 50% completing four to six cycles of chemotherapy. Ivor–Lewis oesophagectomy was the most common surgical approach (55%), followed by total gastrectomy (26%). In 16 patients (52%), surgical treatment of the liver metastases was limited to a single wedge resection. Further details on systemic and surgical treatment are presented in Table 2 and Table S1. A radical resection (R0 resection) was achieved in 25 patients (81%), including patients with an R0 resection of the primary tumour and successful ablation of the liver lesion. Radical treatment was achieved in 8 of the 11 (73%) patients with multiple liver metastases. One patient (3%) showed a pathological complete response of the primary tumour (TRG 1) and liver metastasis after neoadjuvant therapy. Pathological outcomes are shown in Table 3.

### 3.2. Surgical Outcomes

No patients died within 30 days after surgery. In 17 patients (55%), complications occurred and five of these required re-interventions. Pulmonary and infectious complications were most common, both occurring in 19% of all patients. The median length of hospital stay was 11 days [IQR 8–15]. Details on complications and mortality are shown in Table 3.

### 3.3. Overall and Disease-Free Survival

**Figure 1:**
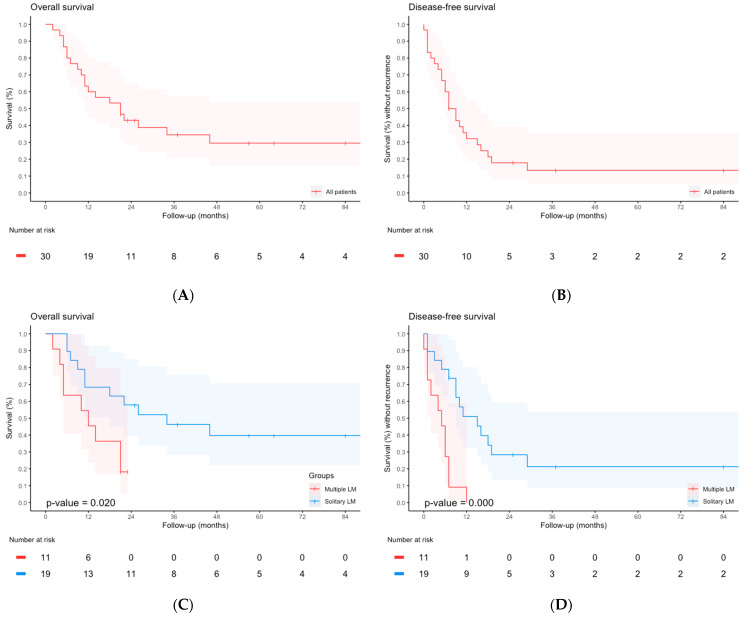
(**A**) Overall survival with 95% CI; (**B**) Disease-free survival with 95% CI; (**C**) Overall survival stratified for number of LM with 95% CI; (**D**) Disease-free survival stratified for number of LM with 95% CI. The *p*-values displayed were calculated with the log-rank test.

One patient was lost to follow-up and was excluded from survival analysis. The median follow-up was 64 months [IQR 37–104]. Median OS was 21 months [IQR 9–36] with 1-, 2-, and 5-year OS rates of 60%, 43%, and 30%, respectively (Figure 1). Median OS was 34 months [IQR 11-not reached] for patients with a solitary liver metastasis, and 12 months [IQR 5–21] for patients with multiple liver metastases (*p* = 0.02). In multivariable Cox regression analysis, solitary liver metastasis was associated with better OS (aHR 0.330 [95% CI 0.125–0.970], *p*-value = 0.025), as shown in Table 4. No other factors were significant in univariable analysis (Table 4).

Median DFS was 7 months [IQR 4–16] with 1-, 2-, and 5-year DFS rates of 31, 18, and 13%, respectively. Median DFS was 15 months [IQR 7–16] for patients with a solitary liver metastasis, compared to 5 months [IQR 1–7] for patients with multiple liver metastases (*p* < 0.001, Figure 1D). Twenty patients had recurrence after complete radical resection (20/25; 80%), of which 19 relapsed within 2 years. In 8 of 25 patients with recurrence (32%), the liver was the first site of recurrence.

## 4. Discussion

This study assessed outcomes after surgical treatment of OGC patients with synchronous liver metastases. Median OS was 21 months [IQR 9–36] with a 2-year survival rate of 43%, no 30-day mortality, and a postoperative complication rate of 55%. Although disease recurred in 80% of patients after complete radical resection, long-term survival was achieved in some patients. Patients with solitary liver metastasis had the best prognosis with a median OS of 34 months. When compared to the survival of patients treated with palliative chemotherapy or best supportive care, these outcomes seem favourable.

In contrast to patients with gastric cancer, no studies have investigated the outcomes of metastasectomy (or ablation) with surgical resection in oesophageal cancer patients with synchronous liver metastases. The median OS following palliative chemotherapy for patients with metastatic (or recurrent) oesophageal or gastric cancer is respectively 6.7 and 11 months, as reported in two Cochrane reviews, both markedly less than the 21 months we describe [3,5]. A recent Dutch nationwide study reported a median OS of 5.7 months for OGC patients with OMD of the liver (defined as ≤ 3 lesions), with only two patients (3.9%) undergoing surgical resection with metastasectomy [21]. Nearly 68% of patients with OMD received best supportive care. Although the inclusion period from this study ended just before the publication of the AIO-FLOT3 trial, it shows that only a small number of patients with OMD of the liver are considered eligible for surgical treatment. However, several case series reported long-term survival after resection of the primary tumour and liver metastases in oesophageal cancer patients [12,13,14,15]. In a cohort with metastatic disease not limited to the liver, a median OS of 13.6 months was reported following induction chemotherapy and resection of the primary tumour and metastatic site [22]. However, no adjuvant therapy was given, the burden of metastatic disease was not described, and a substantial number of patients progressed on neoadjuvant therapy, which all may explain the difference in OS compared to our data.

Patients who respond to neoadjuvant therapy have a better prognosis, irrespective of surgical treatment [23,24,25]. Effective upfront systemic therapy could prevent further dissemination and, even though response serves as a surrogate for tumour biology, allows for selecting patients who are less likely to develop new distant metastases. A so-called ‘test of time’ can be used as an indicator of tumour behaviour, as synchronous OMD might just represent the tip of the iceberg [26]. Since all but one patient had no progression during neoadjuvant therapy in our study, non-responders were denied curative treatment in the participating centres. Within this context, our outcomes seem comparable to the AIO-FLOT3 trial, which reported a median OS of 22.9 months for patients with OMD from gastric and junctional adenocarcinoma who responded to induction chemotherapy [10]. Similar to this study, they included patients with up to five liver lesions, reporting a median OS of 13.6 months for this subgroup. However, only 11 patients with liver OMD were included and the number of liver metastases was not reported.

Characteristics associated with favourable survival for patients with stage IV disease (not limited to the liver) from oesophageal cancer have been described before. Rather than the number of metastases, Blank et al. [22] reported that the resection margin and histopathological response were predictive for OS. Many reports on gastric cancer found the number of liver metastases to be the most important predictor for OS, which is in line with our results [27]. Even though our data suggest that the resection margin was not a significant predictor of OS, positive margins should be avoided since no patient with R1/R2 resection survived for more than 2 years.

An important aspect to consider when submitting metastatic patients to surgical treatment is postoperative mortality and morbidity. Patients undergoing an oesophagectomy are burdened by a higher rate of complications and mortality compared to those undergoing a gastrectomy [28]. In this study, no 30-day mortality was seen and 55% of patients had postoperative complications, with five (16%) requiring re-intervention. This is comparable to large cohort studies for open or hybrid oesophagectomies, reporting around 50–60% postoperative complications and up to 5% mortality [28]. Anastomotic leakage and pneumonia rates were comparable, occurring in respectively 7% and 19% in this study versus 7–10% and 16–19% in previous reports [28,29], and one re-intervention was necessary for bile leakage after liver surgery. Although these rates seem acceptable when compared, it must be considered that the included patients are highly selected since metastasectomy is not the standard of care, reflected by the majority of patients being fit at baseline (WHO 0–1) and a median age of 62 years. More importantly, the quality of life (QoL) of these patients has not been reported, whilst the metastatic setting emphasises the value of QoL.

Although this study showed that prolonged OS can be achieved with surgical treatment for OMD of the liver, it also highlights its challenges. Despite collecting data from several high-volume centres, the number of patients was limited. This underlines that this cohort is a select group and that true OMD is rare given the high chance of simultaneous spread to the lung, bone, peritoneum, or distant lymph nodes, limiting the ability to identify prognostic factors to select patients who could benefit [30,31]. Moreover, targeted therapies such as nivolumab and trastuzumab have become common as therapeutic agents in a metastatic setting; despite including patients treated up to 2020, only three patients received (neoadjuvant) targeted therapy. The FLOT trial [10] has shifted first-line chemotherapy for metastatic gastric cancer and may result in higher response rates. Recent real-world data, however, suggests clinical equipoise in OS between anthracyclin triplets and FLOT chemotherapy [32]. Furthermore, a uniform definition of OMD had not been established until recently. We included patients with up to five liver metastases, but the recent OMEC Delphi study [7] established bilobar ≤ 2 liver metastases or unilobar ≤ 3 liver metastases as OMD, providing a reference for future studies. Six patients in this study did not fit this definition.

Despite these limitations, this is one of the few western studies and the first study to include patients with liver metastases from oesophageal cancer that investigates the survival of OGC patients with synchronous liver metastases who underwent surgical or ablative therapy with curative intent. Our cohort was relatively homogeneous, as metachronous disease was excluded, most patients received neoadjuvant therapy, and all patients were treated in high-volume centres. Long-term OS seems achievable in patients with solitary liver metastasis, but high-level data are anticipated soon. The RENAISSANCE trial (FLOT5) investigates the potential benefit of responders to chemotherapy ± trastuzumab followed by surgery on OS and QoL in patients with limited-metastatic adenocarcinoma of the stomach and junction [33]. The SURGIGAST trial randomises between the continuation of chemotherapy and surgery following 4 cycles of FLOT with OS as the primary endpoint [34]. The outcomes of these RCTs will determine whether surgery has a future role in the treatment of OMD (of the liver), perhaps also clearing the way for surgical management in oesophageal cancer patients.

## 5. Conclusions

In conclusion, long-term survival can be achieved in select patients with synchronous liver metastases from OGC. However, disease recurred in 80% of patients with radical resection of the primary tumour and liver metastases. While surgery may be considered in patients with solitary liver metastasis, as they seem to have a favourable prognosis, more data are needed to identify patients who are likely to benefit from surgical therapy and establish its role as a legitimate treatment option.

## Figures and Tables

**Table 1:** Baseline characteristics.

	Total
Variables	n = 31
**Age, years (median [IQR])**	62 [53–68]
**Gender**	
Male	25 (81)
Female	6 (19)
**WHO performance status**	
0–1	20 (65)
2–3	2 (7)
Missing	9 (29)
**ASA score**	
ASA 1–2	18 (58)
ASA 3–4	13 (42)
**Charlson comorbidity index** **†** **, mean (SD)**	8 (1.75)
**Number of active smokers**	4 (13)
**Tumour location**	
Upper/middle oesophagus	1 (3)
Lower oesophagus/junction	19 (61)
Cardia/proximal stomach	23 (23)
Distal stomach	4 (13)
**Histology**	
Intestinal (ACA)	22 (71)
Diffuse (ACA)	4 (13)
Squamous cell carcinoma	4 (13)
Missing	1 (3)
**Differentiation grade**	
G1	2 (7)
G2	9 (29)
G3	11 (36)
Missing	9 (29)
**Clinical T-category**	
Tx	1 (3)
T1	0 (0)
T2	1 (3)
T3	27 (87)
T4a	1 (3)
Missing	1 (3)
**Clinical N-category**	
Nx	1 (3)
N0	1 (3)
N1	13 (42)
N2	14 (45)
N3	1 (3)
Missing	1 (3)
**Number of LM (median [IQR])**	1 [1–3]
**Rate of solitary LM**	20 (65)
**Diameter of largest LM in mm (median [IQR])**	14 [8–18]
**Intrahepatic distribution of LM**	
Unilobar	28 (90)
Bilobar	3 (10)
**Time of diagnosis LM**	
During surgery (intraoperative)	6 (19)
Clinical staging (preoperative)	25 (81)

IQR, interquartile range; WHO, World Health Organization; ASA, American Society of Anesthesiologists; LM, liver metastasis; ACA, adenocarcinoma. Percentages may not total to 100% due to rounding. † Including 6 points for metastatic disease.

**Table 2:** Systemic and surgical treatment details.

	Total
Variables	n = 31
**Neoadjuvant treatment**	
No	5 (16)
Yes	26 (84)
**Type of neoadjuvant treatment, n = 26**	
Chemoradiation	2 (8)
Chemotherapy	20 (77)
Chemotherapy + targeted therapy	3 (12)
Unknown	1 (4)
**Number of cycles completed**	
3	6 (23)
4–6	13 (50)
>6	4 (15)
Unknown	3 (12)
**Type of resection**	
McKeown esophagectomy	1 (3)
Ivor–Lewis esophagectomy	17 (55)
Transhiatal esophagectomy	2 (7)
Total gastrectomy	8 (26)
Subtotal gastrectomy	3 (10)
**Total minimally invasive procedure**	3 (10)
**Extent of abdominal lymphadenectomy**	
D1	2 (7)
D1+	5 (16)
D2	20 (65)
D3	4 (13)
**Procedures on LM**	
Wedge resection	16 (52)
Multiple wedge resections	4 (13)
Segmentectomy	4 (13)
Ablation	3 (10)
Wedge resection + ablation	4 (13)
**Adjuvant treatment**	
No	17 (55)
Yes	14 (45)
**Type of adjuvant treatment, n = 14**	
Chemoradiation	1 (7)
Chemotherapy	9 (64)
Chemotherapy + targeted therapy	4 (29)
**Number of cycles completed**	
3	6 (43)
4–6	5 (36)
Unknown	3 (21)

LM, liver metastasis; Percentages may not total to 100% due to rounding.

**Table 3:** Pathological outcomes and mortality and morbidity rates.

	Total
Variables	n = 31
**Pathological T-stage**	
pT0	2 (7)
pT1	3 (10)
pT2	3 (10)
pT3	16 (52)
pT4a	5 (16)
pT4b	2 (7)
**Pathological N-stage**	
pN0	3 (10)
pN1	8 (26)
pN2	14 (45)
PN3	6 (19)
**Pathological M-stage**	
pM0	3 (10)
pM1	24 (77)
Not reported	4 (13)
**Number of lymph nodes harvested (median [IQR])**	29 [17–49]
**Number of positive lymph nodes (median [IQR])**	3 [1–6]
**Radicality primary tumour**	
R0 ^†^	28 (90)
R1	2 (7)
R2	1 (3)
**Radicality LM**	
R0	20 (65)
R1	2 (7)
R2	1 (3)
Not applicable (ablation)	8 (26)
**R0 resection rate multiple LM**	8/11 (73)
**Tumour regression grade (TRG)** ^‡^	
TRG 1	1 (3)
TRG 2	3 (10)
TRG 3	3 (10)
TRG 4	9 (29)
TRG 5	6 (19)
Missing	4 (13)
Not applicable *	5 (16)
**Mortality**	
In hospital	0 (0)
30-day	0 (0)
**Rate of complications**	
Any complication	17 (55)
Severe complication requiring re-intervention (CD ≥ 3a)	5 (16)
**Length of ICU stay (median [IQR])**	0 [0–2]
**Length of hospital stay (median [IQR])**	11 [8–15]
**Rate of readmission < 30 days**	3 (10)
**Specification of complications** ^	
Rate of pulmonary complications	6 (19)
Rate of cardiac complications	2 (7)
Rate of anastomotic complications	2 (7)
Rate of infectious complications	6 (19)
Rate of gastro-intestinal complications	5 (13)
Rate of other complications	2 (7)

IQR, interquartile range; LM, liver metastasis; TRG, tumour regression grade; CD, Clavien–Dindo classification; ICU, intensive care unit. Percentages may not total to 100% due to rounding. ^†^ R0 was defined tumour-free margins (more than 0 mm). ^‡^ According to Mandard score [19]. * No neoadjuvant therapy given. ^ Defined according to the definitions of the Esophagectomy Complications Consensus Group [20].

**Table 4:** Univariable and multivariable cox regression analysis for overall survival.

	Univariable Analysis			Multivariable Analysis		
Variables	HR	CI (95%)	*p*-Value	aHR	CI (95%)	*p*-Value
**Age, per year**	1.001	0.962–1.040	0.975			
**Differentiation (well-mod vs. poor)**	1.525	0.526–4.423	0.437			
**Lymphovascular invasion (no vs. yes)**	1.647	0.654–4.148	0.290			
**Tumour location (oesophagus vs. stomach)**	0.803	0.323–1.996	0.636			
**Liver metastases**						
Solitary vs. multiple	0.338	0.130–0.955	**0.028**	0.33	0.125–0.870	**0.025**
Diameter, per cm	0.862	0.468–1.588	0.634			
Unilobar vs. bilobar	0.978	0.223–4.287	0.976			
**Neoadjuvant therapy (yes vs. no)**	1.822	0.660–5.029	0.247			
**Time of diagnosis LM (pre- vs. intraoperative)**	1.596	0.578–4.410	0.367			
**Extent of lymphadenectomy (D1-D1+ vs. D2–3)**	0.472	0.188–1.181	0.109	0.455	0.182–1.140	0.093
**Complete radical resection ^†^ (yes vs. no)**	1.935	0.628–5.967	0.250			
**Adjuvant therapy (yes vs. no)**	1.602	0.660–3.889	0.297			
**Complication CD≥3a (no vs. yes)**	1.755	0.406–7.586	0.451			

HR, hazard ratio; aHR, adjusted HR; CI, confidence interval; LM, liver metastasis; CD, Clavien–Dindo score. ^†^ R0 resection of primary tumour and liver metastases or successful ablation. **Bold** values indicate a significant *p*-value (<0.05).

## Data Availability

The datasets used and analysed will be available upon reasonable request.

## Supplementary Materials

The following supporting information can be downloaded at: https://www.mdpi.com/article/10.3390/cancers16040797/s1, Table S1 Systemic treatment details.
