# Supplementary material for: Outcomes after Surgical Treatment of Oesophagogastric Cancer with Synchronous Liver Metastases: A Multicentre Retrospective Cohort Study
Source: Cancers (Basel). 2024 Feb 16;16(4):797. doi: 10.3390/cancers16040797 (PMC10887104; doi:10.3390/cancers16040797)
Supplement: Supplementary file 1 [file cancers-16-00797-s001.zip › cancers-2849783-supplementary.pdf]

Supplement

Table S1 Systemic treatment details stratified per histological subtype

Type of treatment	***Neoadjuvant*** *n = 26*	***Adjuvant*** *n = 14*
Squamous cell carcinoma		
**CRT (CROSS)**	1 (4)	0 (0)
**Chemotherapy**		
Doublet (platinum based)	2 (8)	1 (7)
Triplet (anthracyclins)	1 (4)	0 (0)
Adenocarcinoma		
**CRT** (Doublet, platinum based + 45 Gy)	1 (4)	0 (0)
**Chemotherapy**		
Doublet (platinum based)	9 (35)	5 (36)
Triplet (anthracyclins)	6 (23)	1 (7)
FLOT	3 (12)	2 (14)
**Chemotherapy + targeted therapy**		
Doublet (platinum based) + trastuzumab	1 (4)	2 (14)
Monotherapy + trastuzumab	1 (4)	1 (7)
**Trastuzumab**	0 (0)	1 (7)
**Unknown**	1 (4)	1 (7)

CRT Chemoradiotherapy *Gy* Gray
